# Suspected Stevens-Johnson Syndrome After Azithromycin and Doxycycline Exposure in an Elderly Woman: A Case Report Showcasing Treatment Based on Clinical Diagnosis

**DOI:** 10.7759/cureus.105223

**Published:** 2026-03-14

**Authors:** Madhumita Baskaran, Anna Kim, Wilfredo Grana

**Affiliations:** 1 Department of Research, Alabama College of Osteopathic Medicine, Dothan, USA; 2 Internal Medicine, Regional Medical Center, Anniston, USA

**Keywords:** azithromycin, doxycycline, drug-induced skin reaction, skin reaction, stevens-johnson syndrome (sjs), toxic epidermal necrolysis (ten)

## Abstract

Drug-induced cutaneous reactions can range from mild eruptions to life-threatening syndromes such as Stevens-Johnson syndrome (SJS), often presenting a diagnostic and management challenge. We present the case of a 76-year-old woman who developed a rapidly progressive, diffuse erythematous rash with systemic manifestations following sequential use of two different antibiotics for the treatment of unresolving cold symptoms. Due to the complexity of definitive medical diagnosis of severe drug-induced hypersensitivity reactions and histopathological access, treatment relied on clinical diagnosis based on findings, patient history, and symptoms. Prompt discontinuation of suspected medications and initiation of multidisciplinary supportive care resulted in significant clinical improvement, with the patient returning close to baseline prior to hospital discharge. This case highlights the diagnostic complexity of antibiotic-associated reactions and underscores the importance of early recognition and management to prevent disease progression and recurrence.

## Introduction

Stevens-Johnson syndrome (SJS) and toxic epidermal necrolysis (TEN) represent a spectrum of severe, life-threatening drug-induced hypersensitivity reactions characterized by mucocutaneous erosions, epidermal detachment, and systemic involvement. SJS is defined as less than 10% body surface area (BSA) detachment and TEN as greater than 30% BSA involvement [1]. The clinical presentation of SJS/TEN typically begins with a prodrome of fever and influenza-like symptoms, subsequently followed by the development of painful, erythematous skin lesions that progress to blistering and epidermal detachment. Eighty percent (80%) of cases show mucosal involvement, particularly affecting the oral cavity, eyes, genitalia, and respiratory tract [2]. Other systemic manifestations can include hepatic dysfunction, renal impairment, and gastrointestinal involvement. Diagnosis is primarily clinical, based on characteristic skin findings, timeline from drug exposure, and histopathologic analysis showing full-thickness epidermal necrosis [1].

Antibiotics are known to be the most common triggers for SJS/TEN, specifically for approximately 28-35% of all cases. Of the antibiotic classes, β-lactams (including penicillins and cephalosporins) represent 39% of antibiotic-associated SJS/TEN, followed by fluoroquinolones and sulfonamides. Other triggering medications include allopurinol, anticonvulsants, and nonsteroidal anti-inflammatory drugs (NSAIDs). Typically, symptoms present four to 28 days after continuous exposure to the antibiotics, although reactions can occur more rapidly if the patient has had previous exposure [1]. 

Cutaneous adverse drug reactions are associated with significant morbidity and mortality due to extensive skin involvement and even multiorgan damage. Early recognition of symptoms and prompt withdrawal of the offending agent, and initiation of supportive and immunosuppressive therapy are critical for improving outcomes. Histopathologic findings may be nonspecific, further complicating treatment. However, diagnosis can be challenging as clinical presentations alone may need to be used for the conclusion. 

The presenting case is of a 76-year-old woman who developed a rapidly progressive diffuse erythematous rash with systemic manifestations after sequential antibiotic exposure, illustrating the diagnostic complexity and management of severe drug-induced hypersensitivity reactions in the elderly. We will dive into the clinical and laboratory findings used to diagnose and treat a patient presenting with a diffuse, systemic skin rash without a known, definitive cause due to the consecutive use of two different antibiotics. However, with the use of clinical judgement, the patient was able to make a significant recovery close to baseline before discharge from the hospital. This highlights the challenges of determining the cause of an adverse reaction and managing it so that symptoms do not worsen or return.

## Case presentation

A 76-year-old Caucasian female with a past medical history of hypertension, hyperlipidemia, mitral valve prolapse, ischemic colitis, macrocytosis, diverticulitis, and external hemorrhoids presented to the emergency department for evaluation of a diffuse rash ongoing for the past five days. On onset, the rash started as a small red dot on her right thigh and has since spread diffusely to her trunk, back, bilateral arms, and upper regions of her bilateral legs the next morning. The rash was associated with a constant moderate-to-severe itching and burning sensation. Associated symptoms include sweating, fatigue, sore throat, and generalized weakness.

Two weeks prior to this visit, the patient was seen by her PCP for cold symptoms, presenting as a sore throat and chest congestion. She was initially started on azithromycin and administered an intramuscular steroid injection. Due to failed treatment with azithromycin, she was subsequently started on doxycycline. The onset of this rash started after completion of both courses of antibiotics. Physical exam reveals diffuse erythematous blanching maculopapular rash to the bilateral upper extremities, trunk, back, and upper regions of bilateral lower extremities, with very minimal below the knees. Rash is with bluish discoloration on her back.

Labs, indicated by Table 1, revealed elevated CRP (152.3 mg/dL) and elevated sedimentation rate (80 mm/h), indicating inflammation. She had an elevated WBC of 17.2 x 10^9^/L with elevated neutrophils of 91.8%, decreased lymphocytes of 2.4%, and no eosinophilia (2.6%). Upon initial presentation, she was also found to have oral candidiasis, leukocytosis, and hyponatremia (likely from dehydration). The treatment plan included immediately discontinuing all antibiotic medications, fluid resuscitation, and supportive treatment for itching and burning. Infectious disease was consulted and recommended maintaining off of antimicrobials and starting on systemic steroids with supportive measures. At this point, the differential diagnosis includes SJS, drug reaction with eosinophilia and systemic symptoms (DRESS) syndrome, erythema multiforme, vasculitis, Still’s disease, or HHV-6. Images of the patient's rash on day 1 were not taken because consent was not yet obtained from the patient.

**Table 1:** Lab values from initial presentation, day 1 of hospitalization.

Lab	Patient's value	Reference range
C-reactive protein (CRP)	152.3 mg/dL	<10 mg/dL
Erythrocyte sedimentation rate (Sed Rate)	80 mm/h	<20 mm/h
White blood cell count (WBC)	17.2 x 10^9/L	5-10 x 10^9^/L
Neutrophils	91.8%	55-70%
Lymphocytes	2.4%	20-40%
Eosinophils	2.6%	1-4%

**Figure 1:**
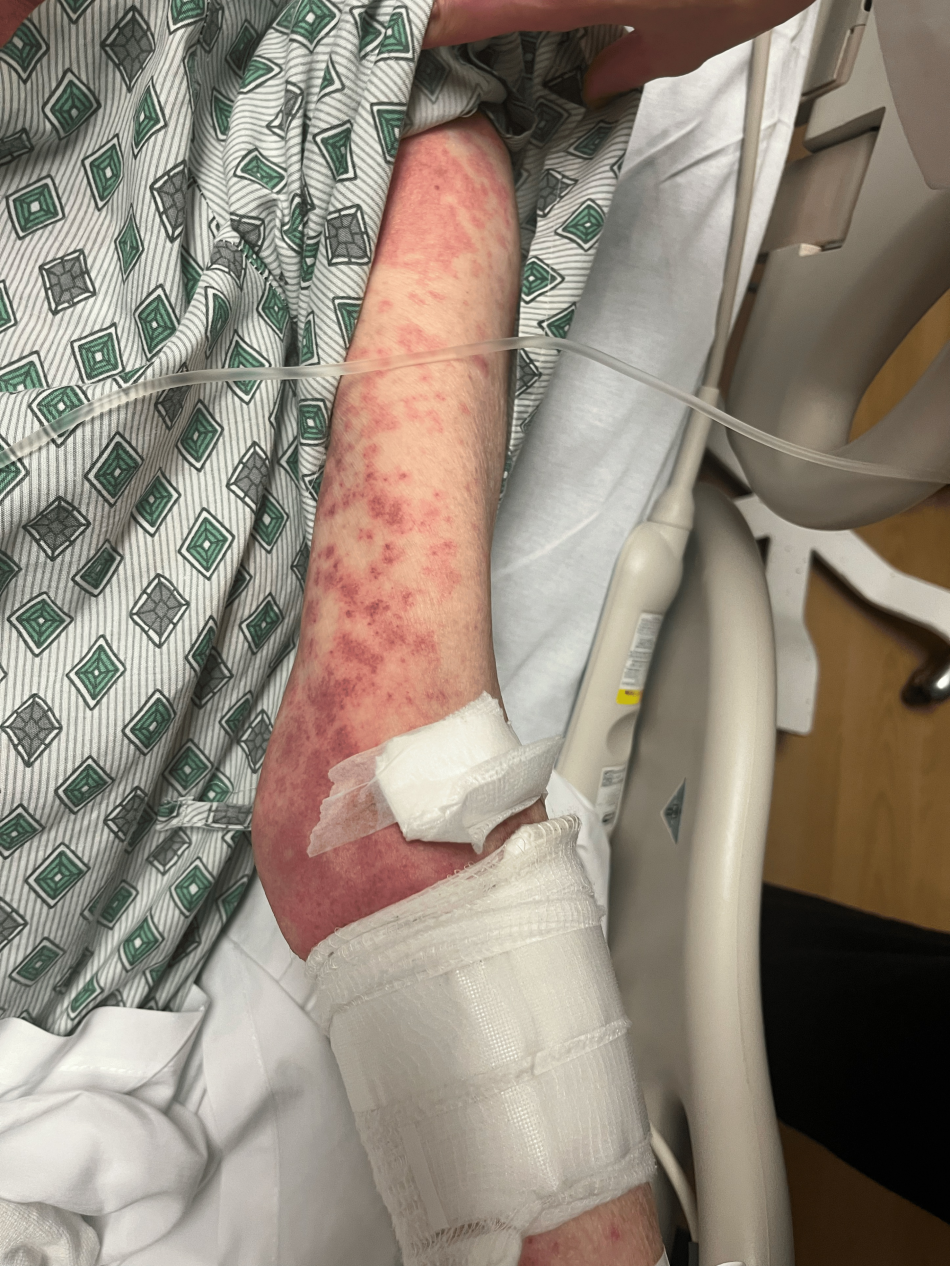
Rash from day 2 (left arm).

**Figure 2:**
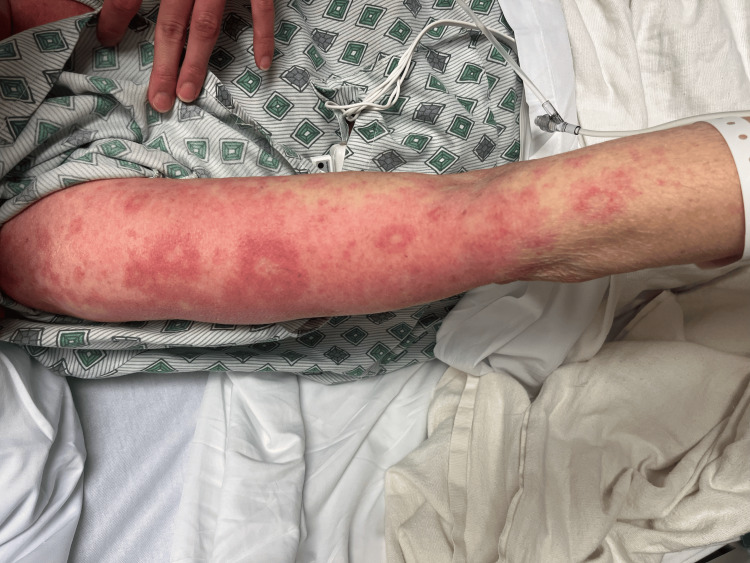
Rash from day 2 (right arm)

**Figure 3:**
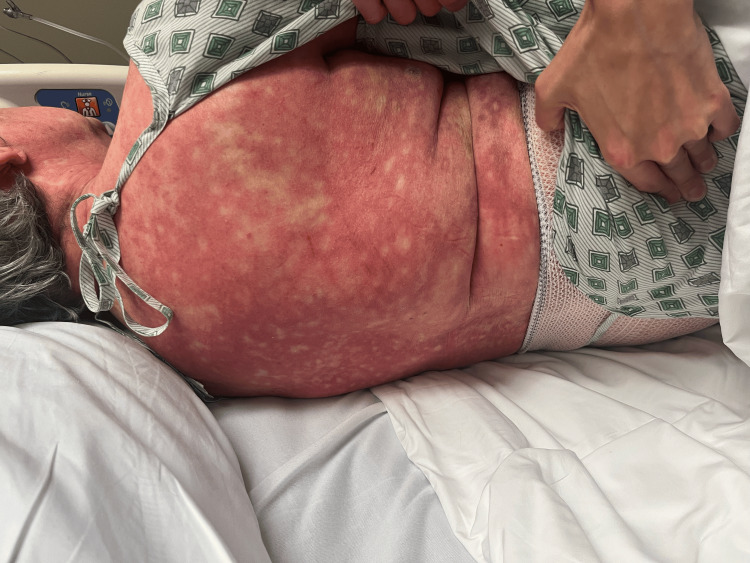
Rash from day 2 (back).

**Figure 4:**
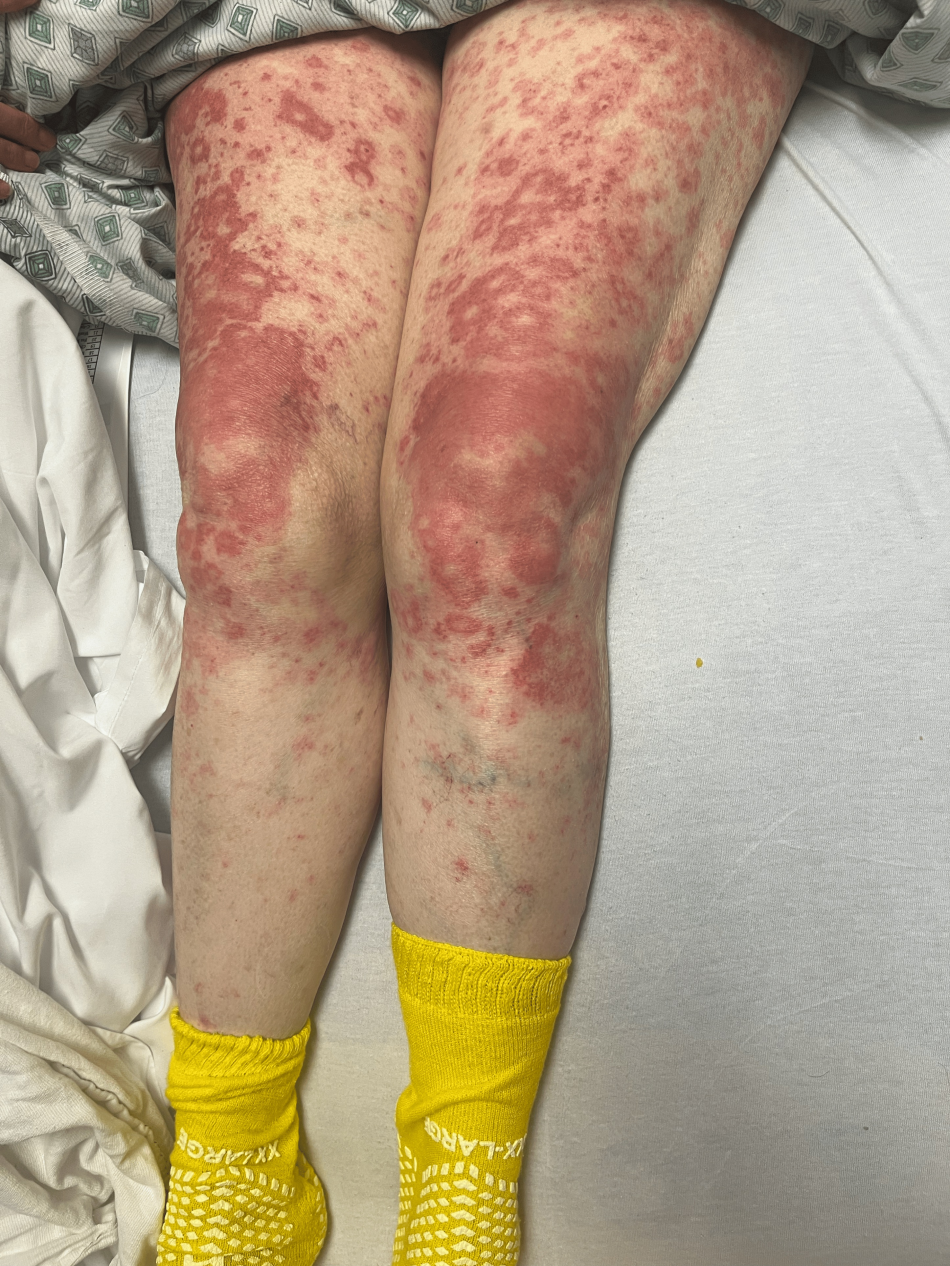
Rash from day 2 (bilateral legs).

**Figure 5:**
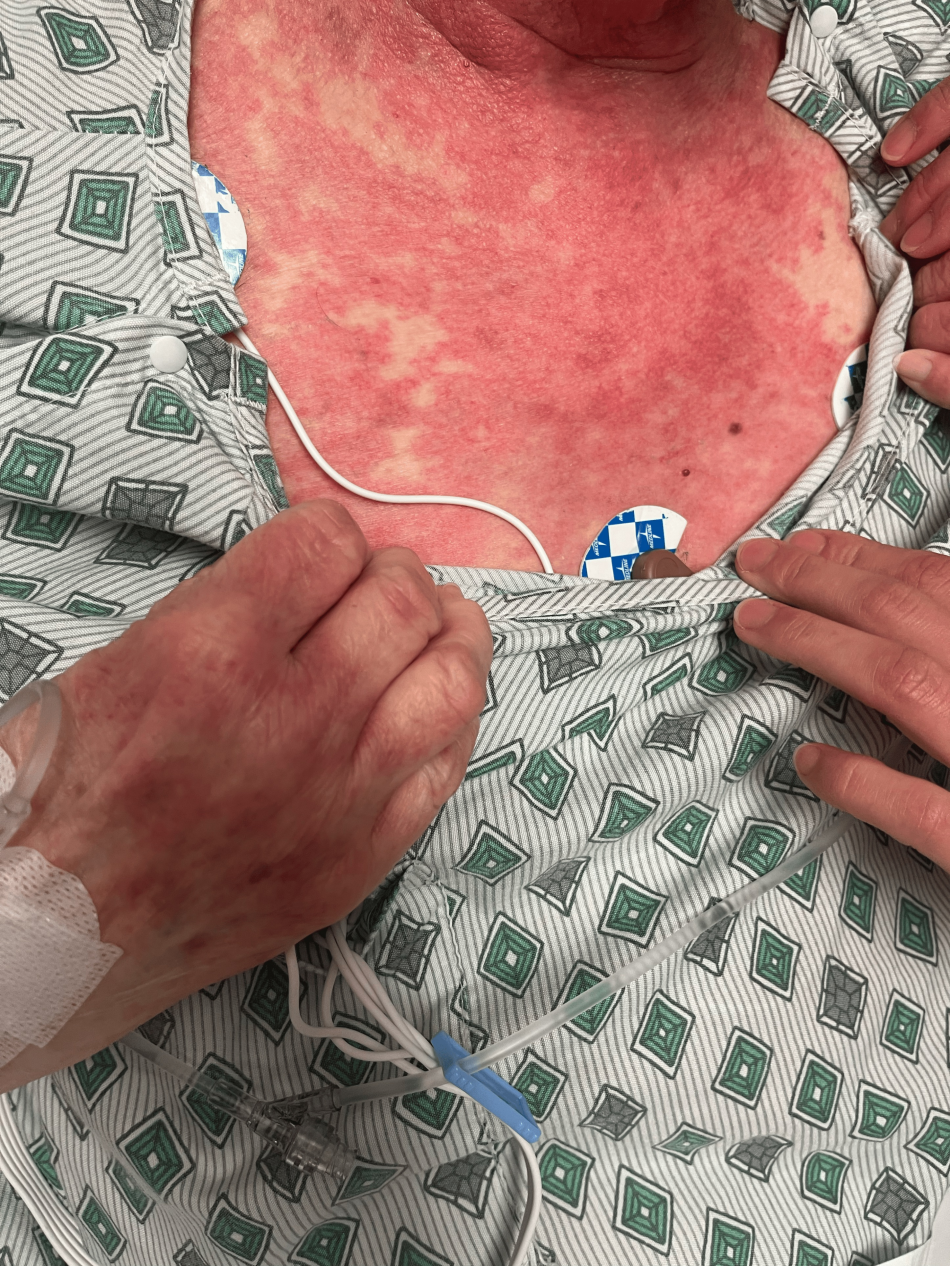
Rash from day 2 (chest).

On day 2, the patient’s rash had spread to below her knees and to her face. The rash is still burning and itching. Topical clobetasol has provided some relief. She denies any nausea, vomiting, fevers, or chills. The patient stated that she has had all of her childhood vaccinations, which rules out any childhood exanthems. Physical exam showed that the same rash had spread to below the knees bilaterally and to the face. Bluish discoloration seen on day 1 has resolved, but erythema is still present. At this point, maintenance of antibiotics, systemic steroids, and supportive therapy was continued. A skin biopsy was performed and sent to pathology for review. Images showing the patient’s rash on this day are shown below (Figures 1-5).

**Figure 6:**
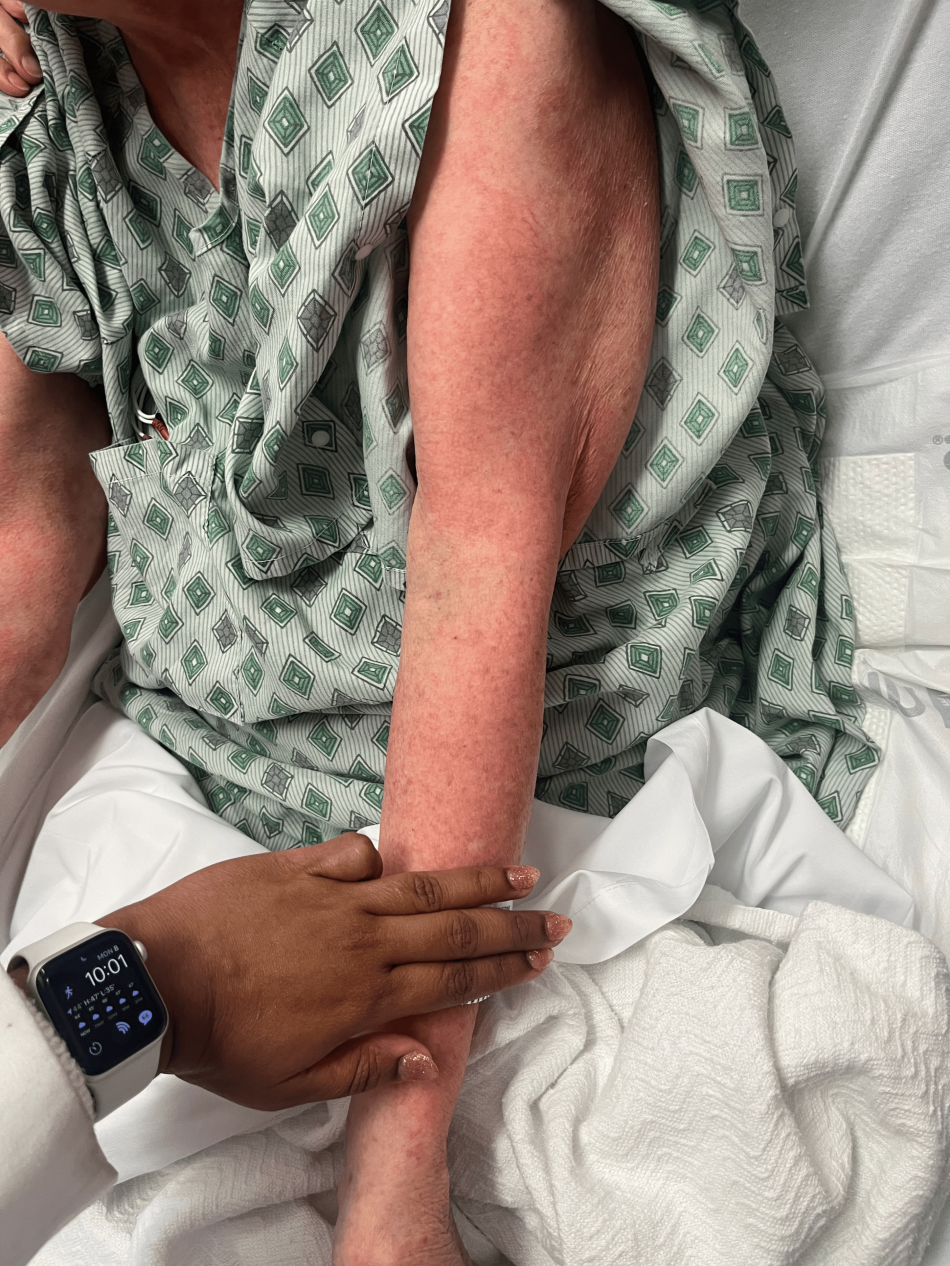
Rash from day 4 (left arm).

**Figure 7:**
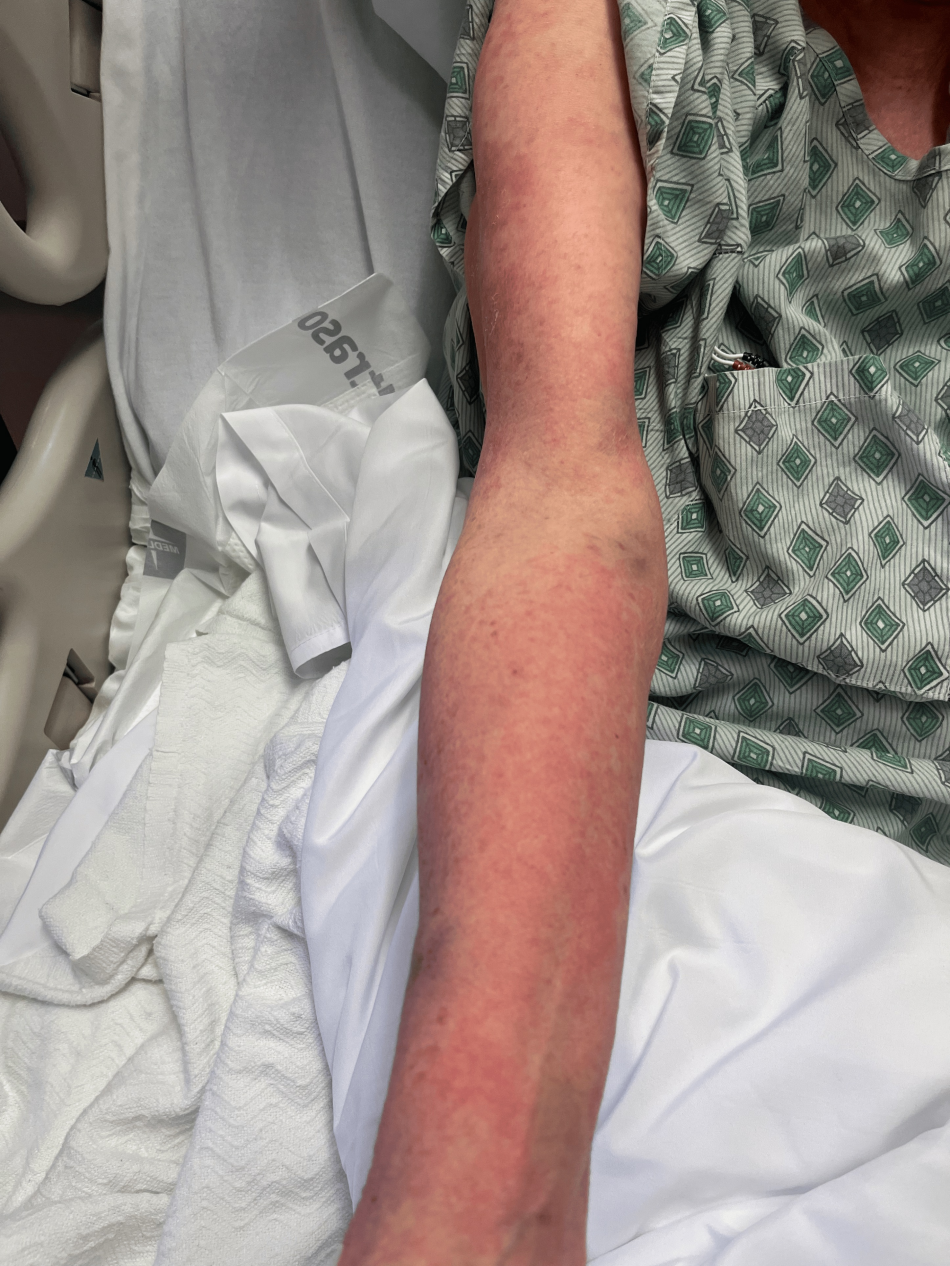
Rash from day 4 (right arm).

**Figure 8:**
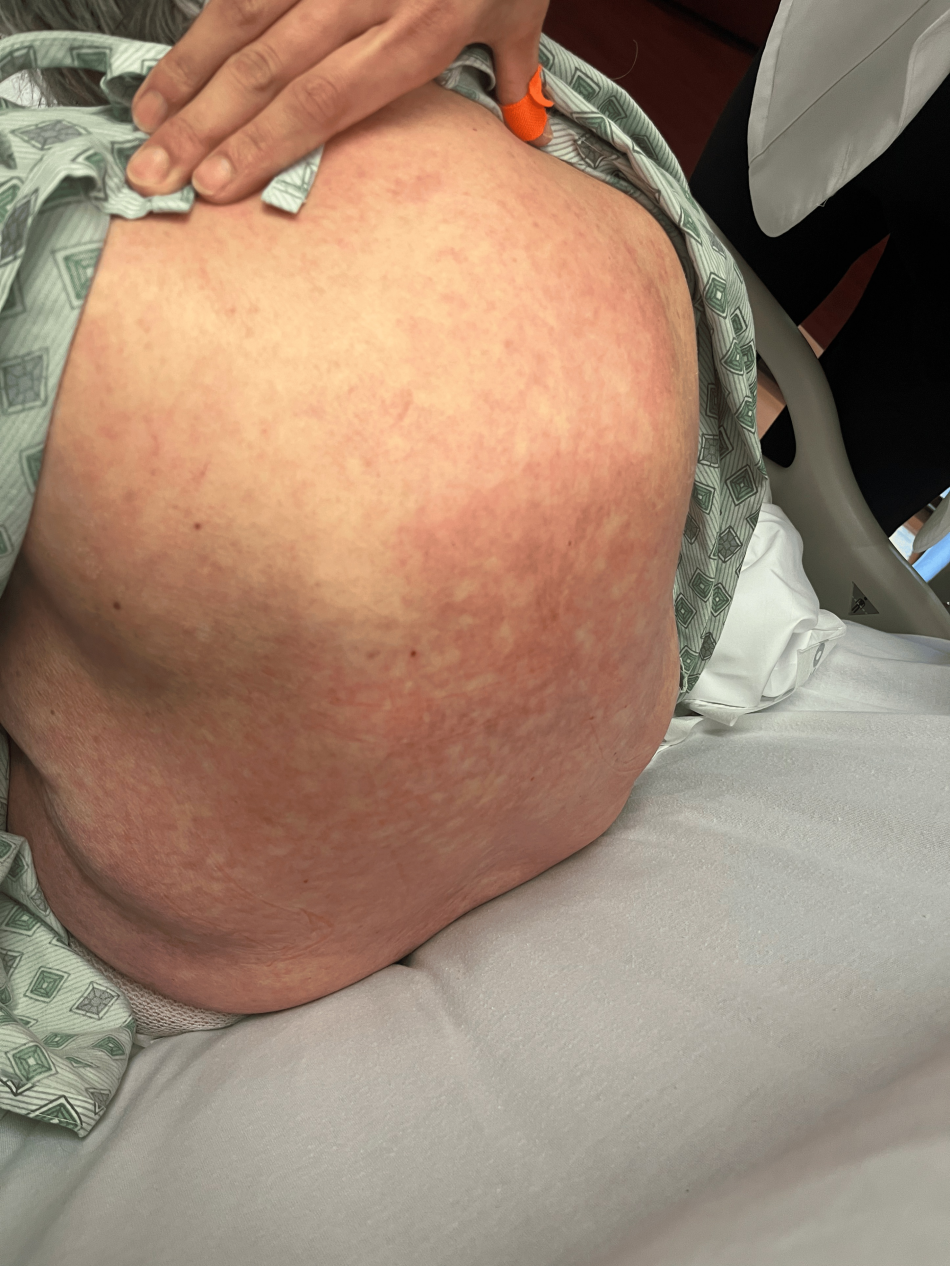
Rash from day 4 (back).

**Figure 9:**
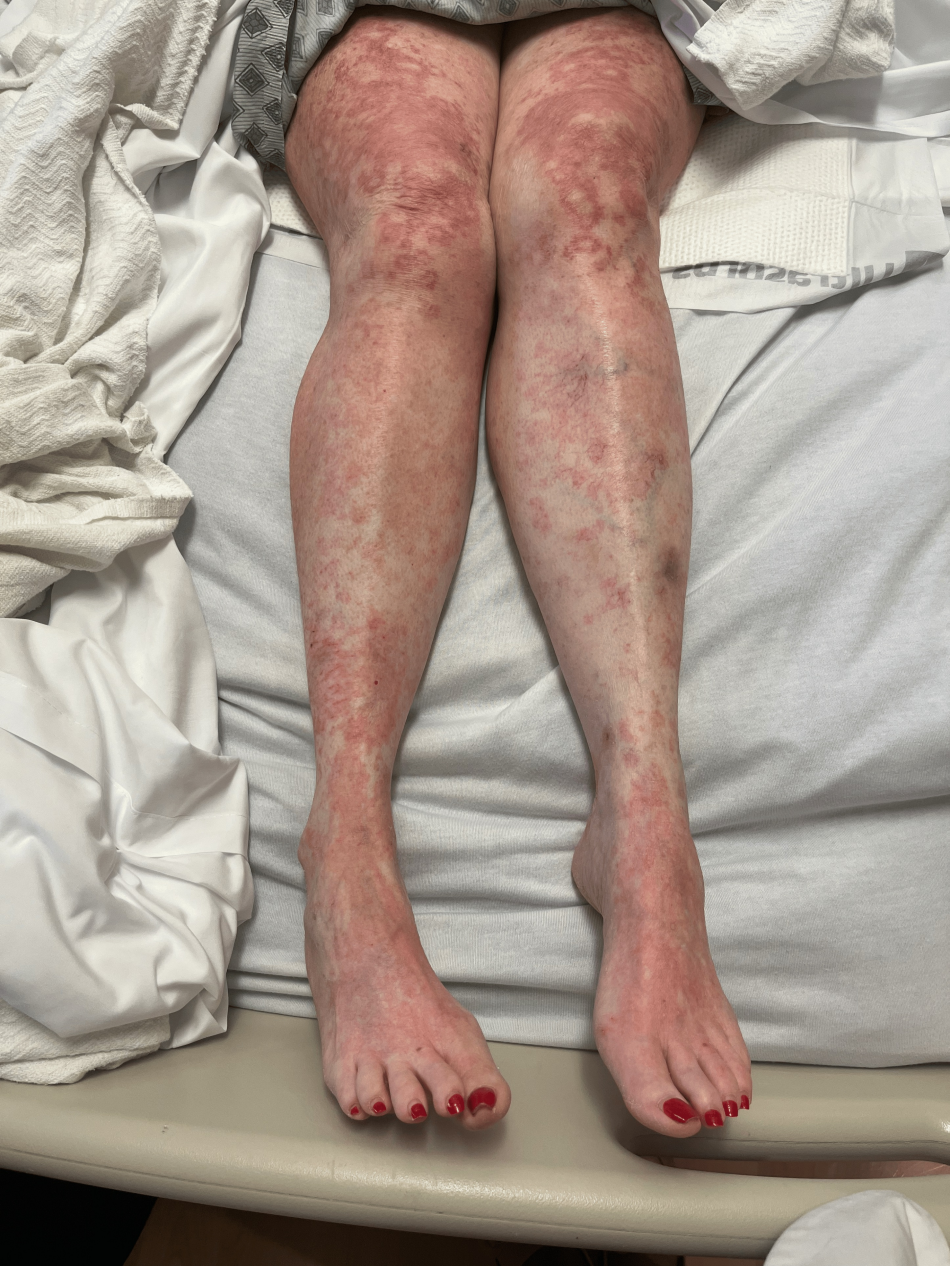
Rash from day 4 (bilateral legs).

**Figure 10:**
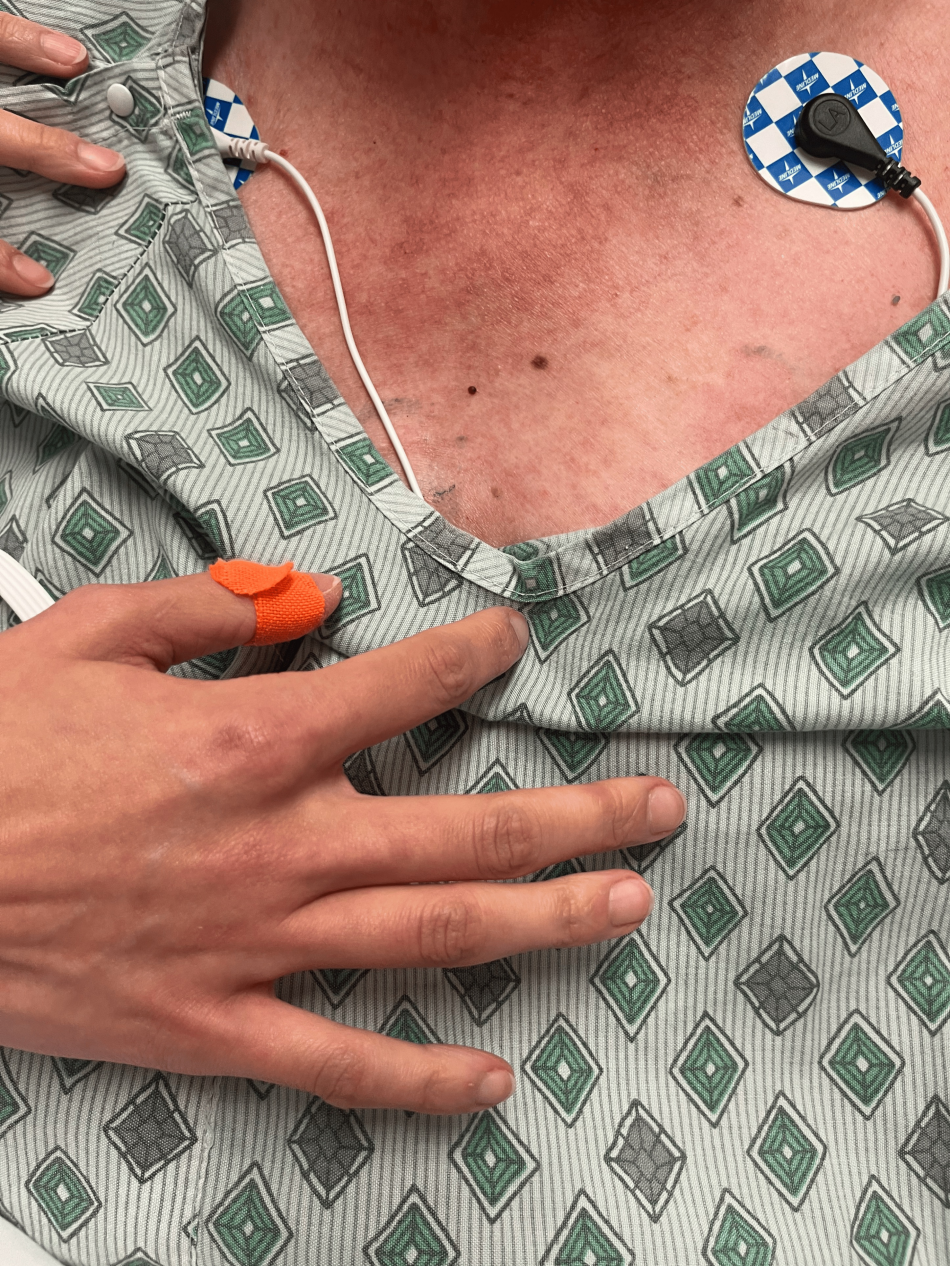
Rash from day 4 (chest).

Over the subsequent 72 hours, the rash progressed to involve the face and distal lower extremities with persistent pruritus and burning. Despite initial worsening, symptoms gradually improved following systemic corticosteroids and topical therapy, with marked clinical improvement of symptoms by day 5. Several antibody markers were tested to evaluate for autoimmune disorders, such as RF, JO-1 antibody, scleroderma antibody, SSA and SSB, SM antibody, and RNP antibody, which were all negative. Images showing the patient’s rash on this day are shown below (Figures 6-10).

**Figure 11:**
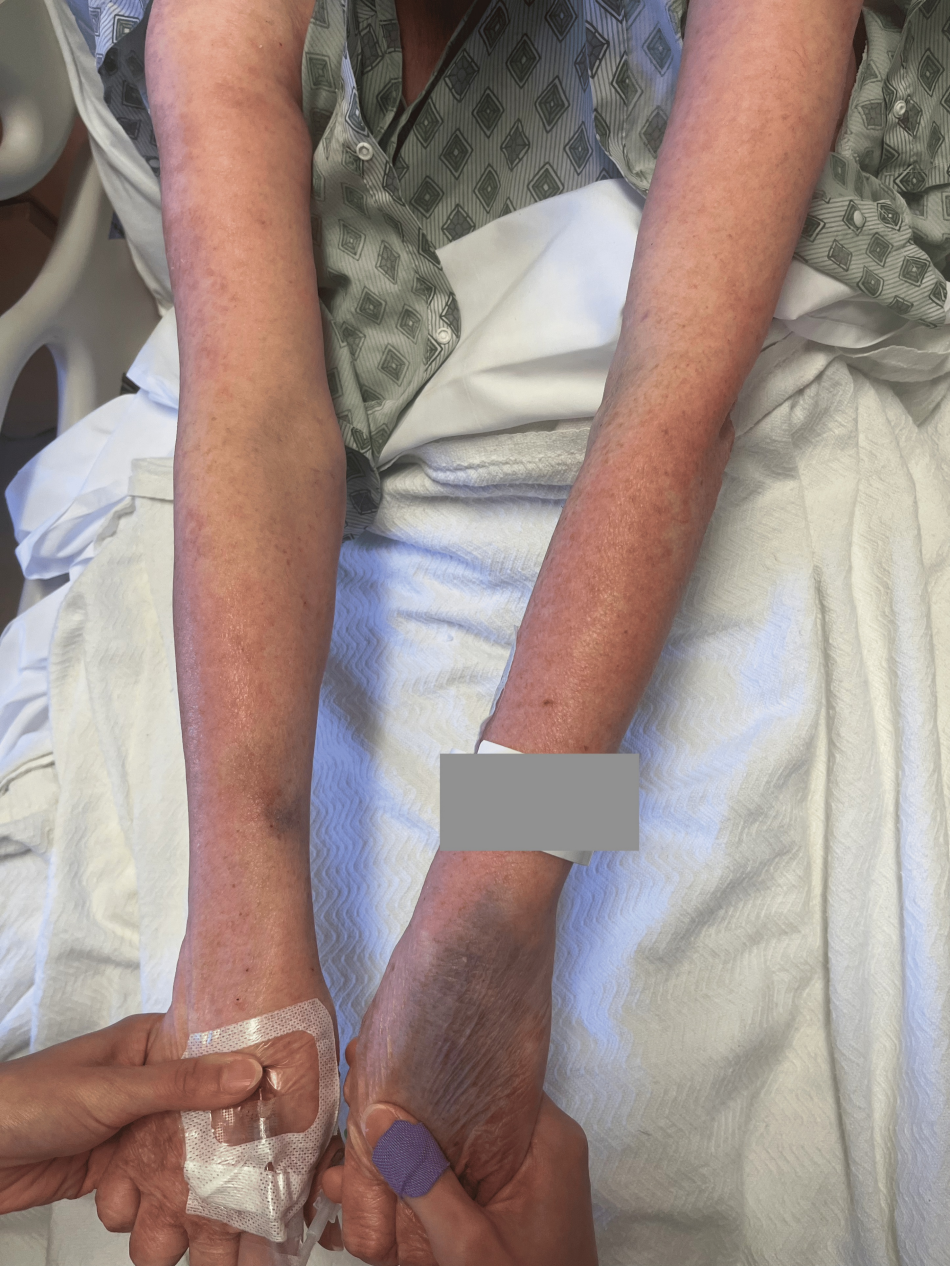
Rash from day 5 (bilateral arms).

**Figure 12:**
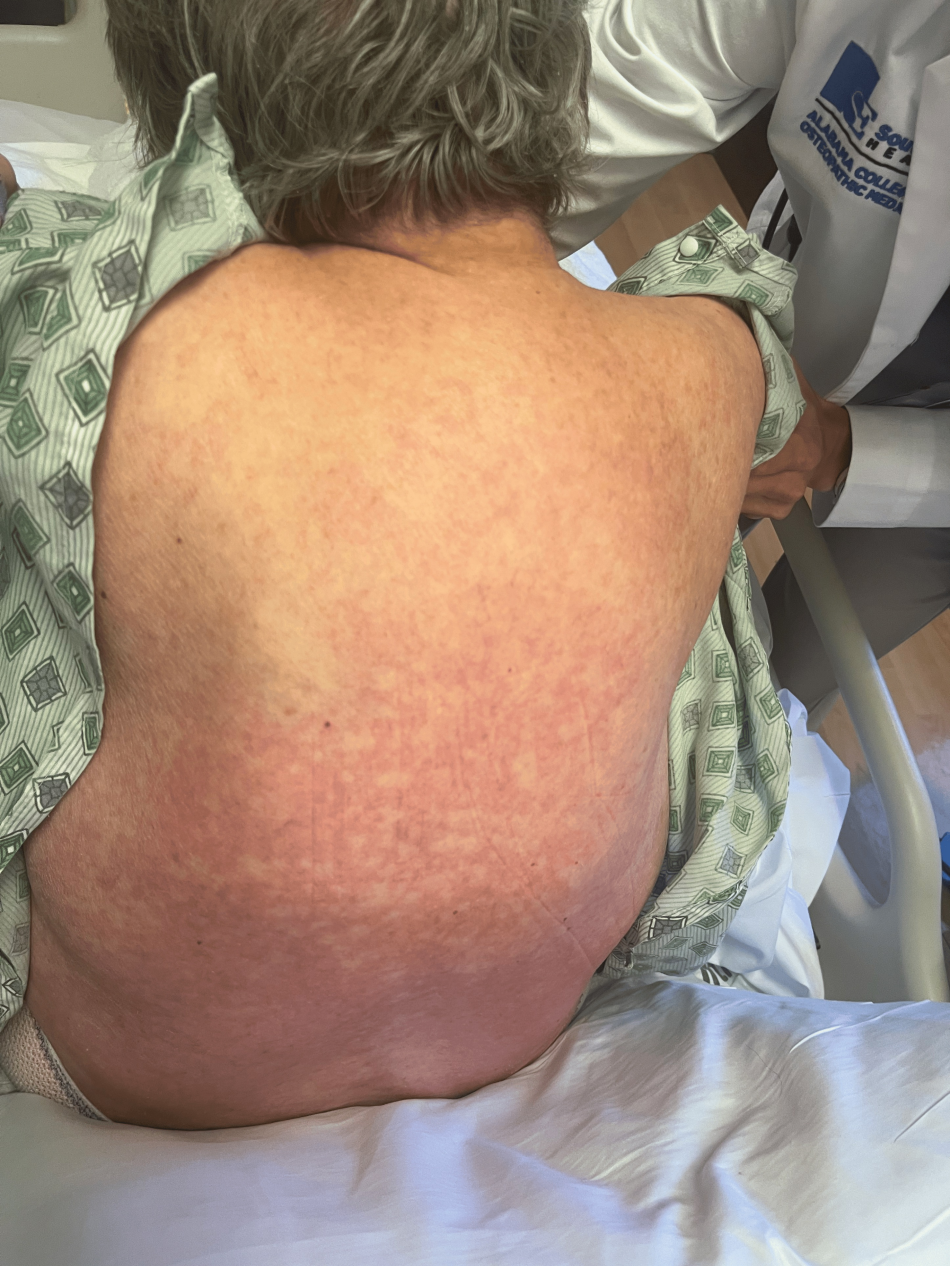
Rash from day 5 (back).

**Figure 13:**
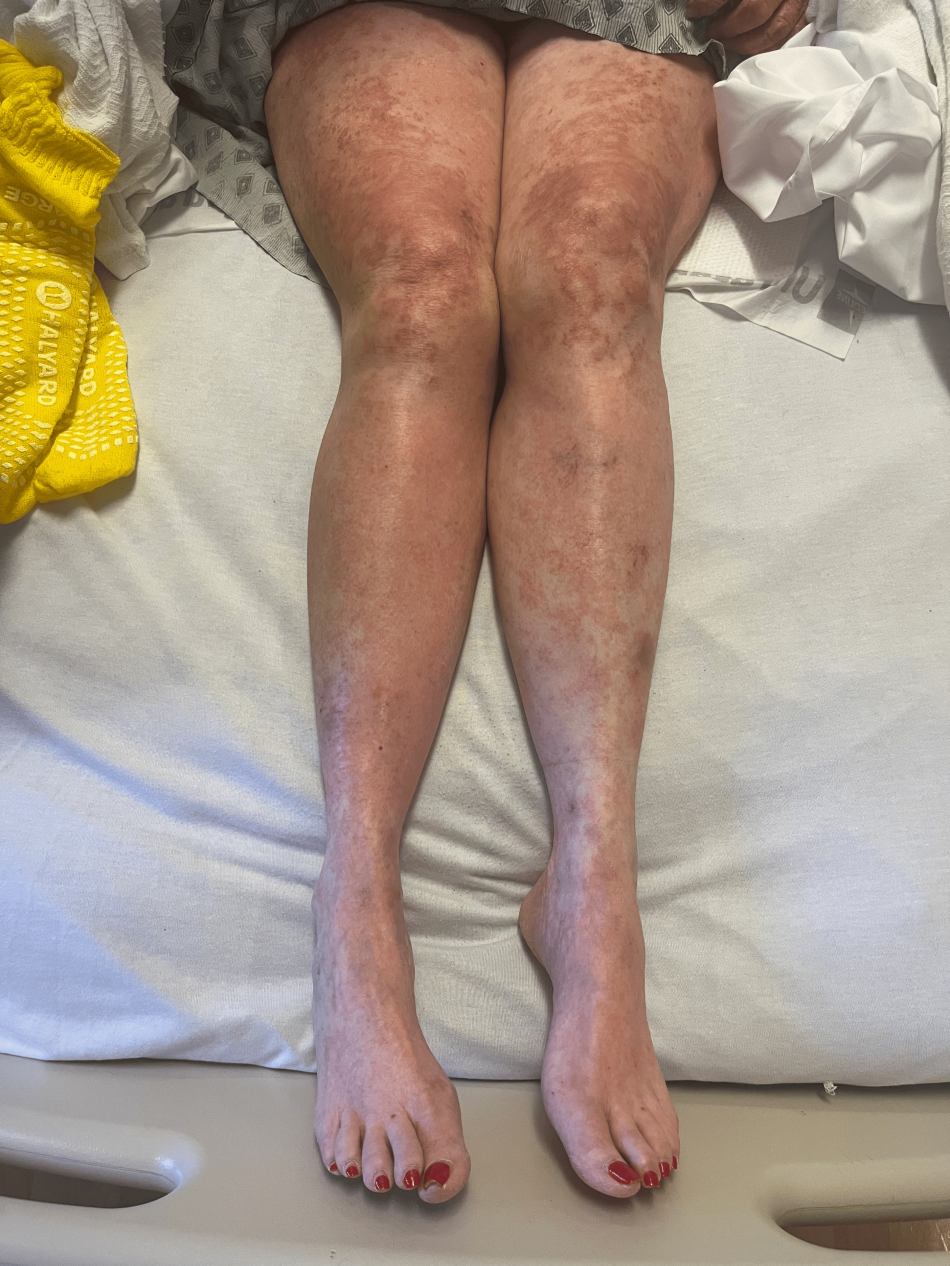
Rash from day 5 (bilateral legs).

**Figure 14:**
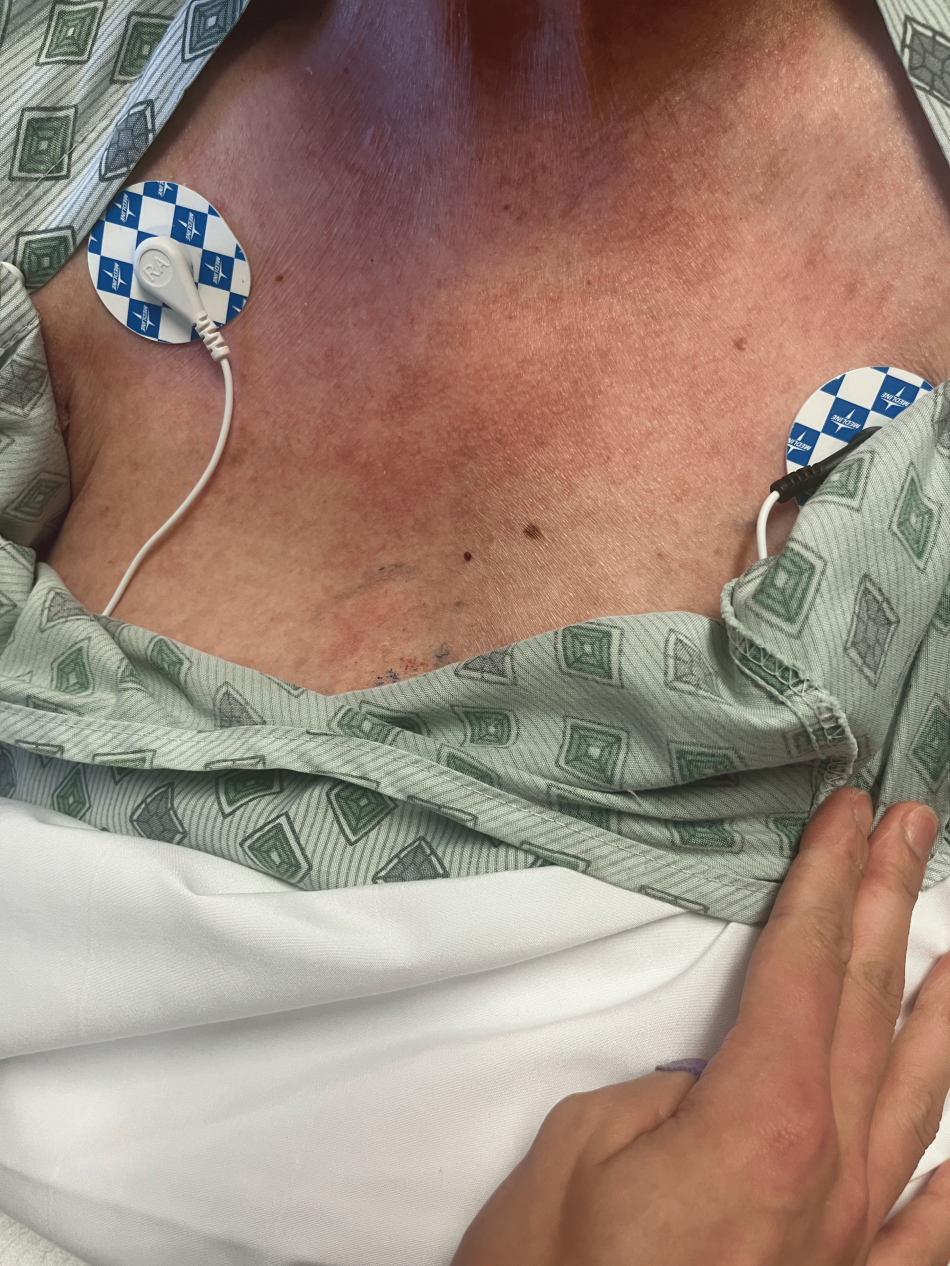
Rash from day 5 (chest).

On day 5, the patient’s symptoms have improved drastically. She is still experiencing a mild itching and burning sensation in her rash. Her rash look improved from the onset of symptoms. The physical exam shows that the rash appeared to be fading diffusely from her body. The preliminary results from the skin biopsy showed benign skin with mild perivascular chronic inflammation. These results are nonspecific, which makes it difficult to conclude a diagnosis from the biopsy. Based on the clinical presentation, this patient likely had SJS. Images showing the patient’s rash on this day are shown below (Figures 11-14).

**Figure 15:**
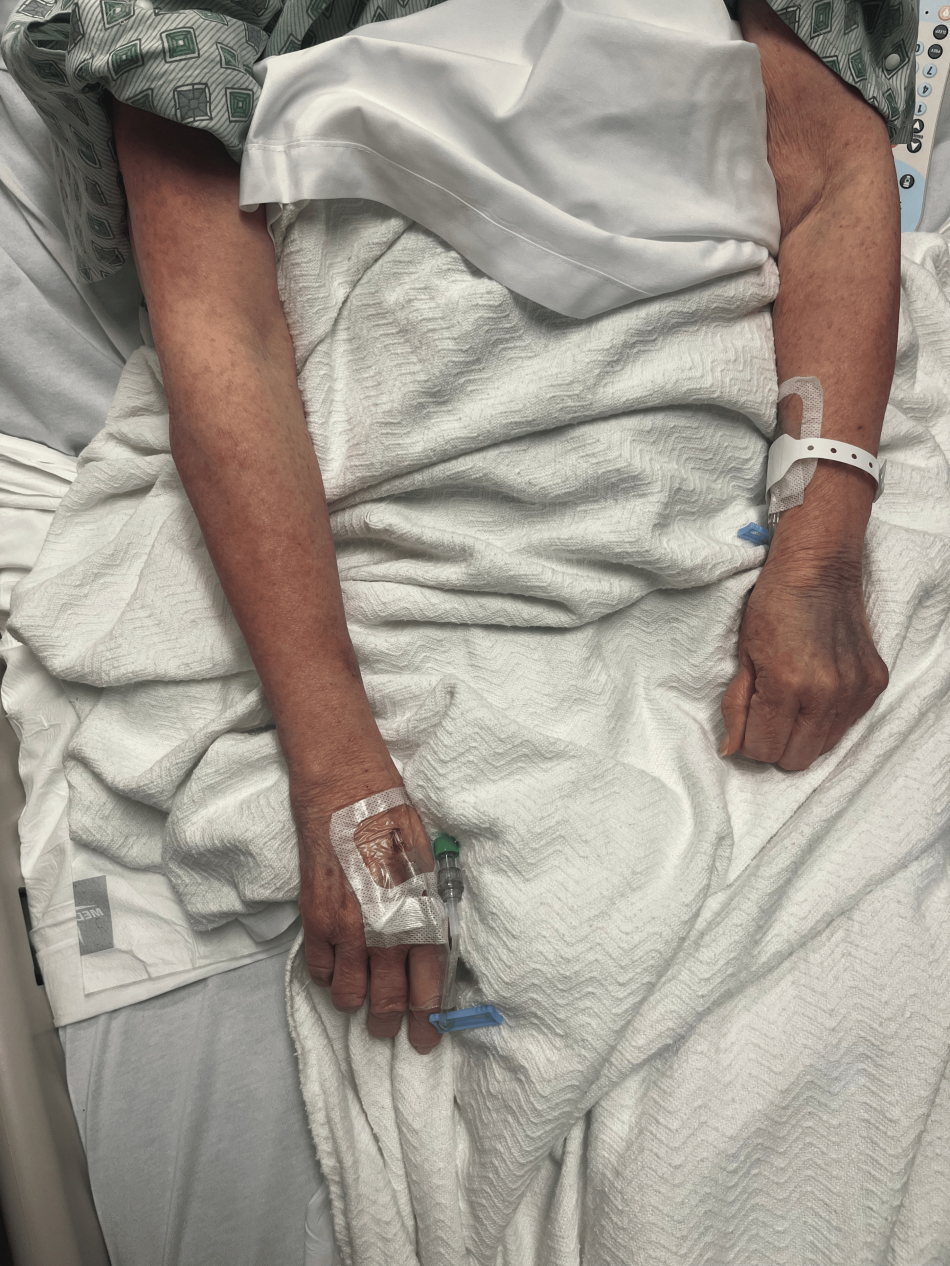
Rash from day 6 (bilateral arms).

**Figure 16:**
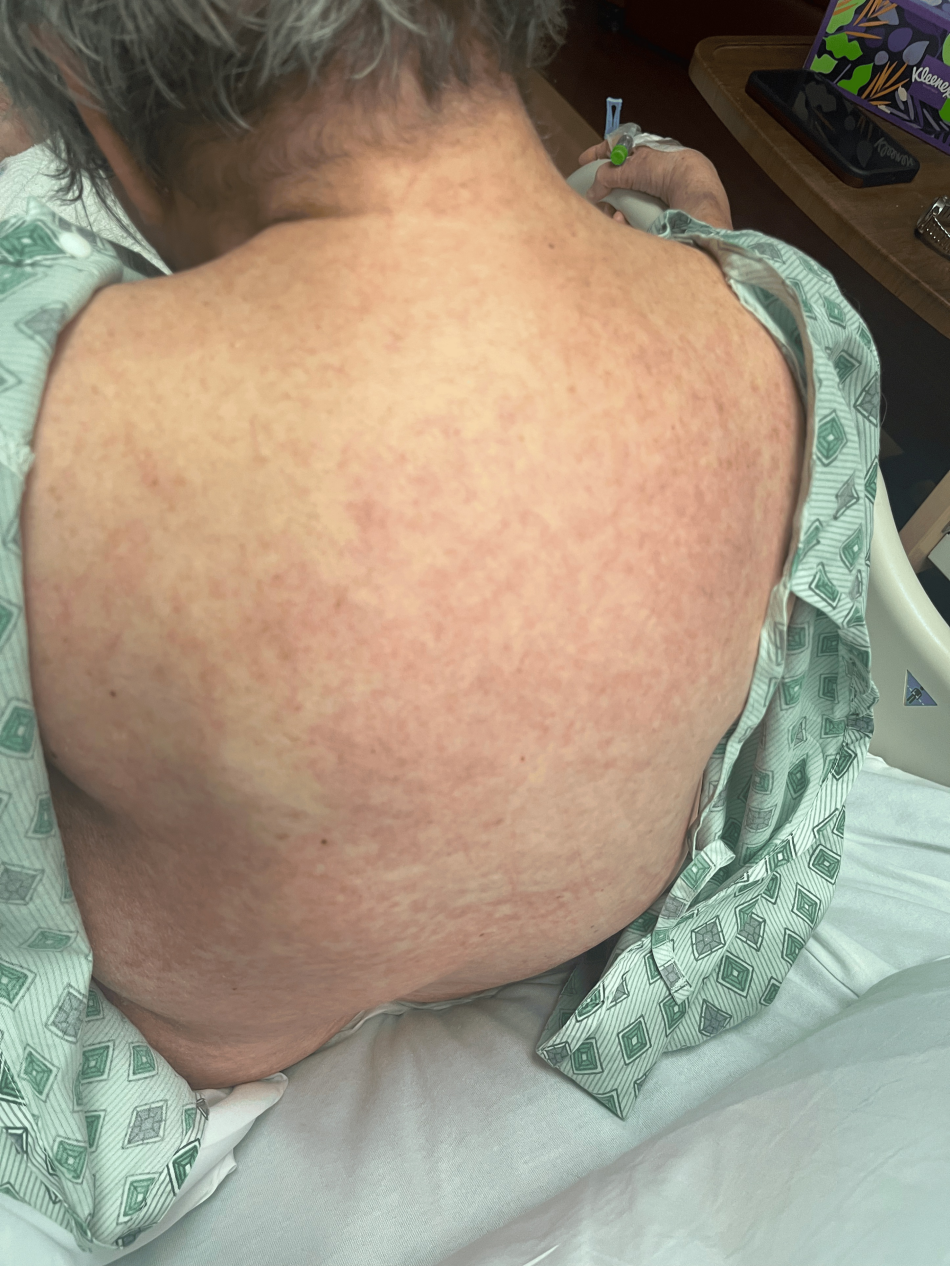
Rash from day 6 (back).

**Figure 17:**
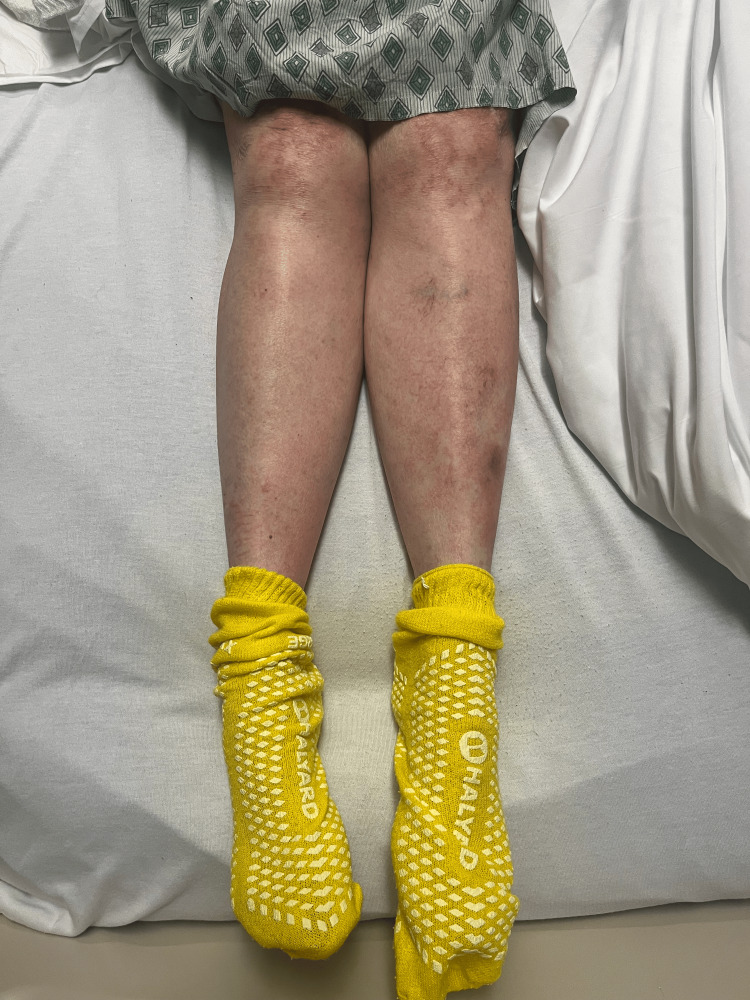
Rash from day 6 (bilateral legs).

By day 6, significant improvement of symptoms and clinical findings was noted. She was discharged with instructions to avoid azithromycin and doxycycline. Final pathology results of the biopsy are pending. The patient was discharged on a tapering dose of her systemic steroids and prescription of her topical steroids of clobetasol and triamcinolone cream. Images showing the patient’s rash on this day are shown below (Figures 15-17).

Weeks later, after the patient was discharged, the final biopsy results indicated a dermal hypersensitivity reaction. Although the biopsy was not conclusive for SJS, a clinical diagnosis needed to be made, further indicating SJS, even though the patient did not have mucosal involvement. This diagnosis was made based on the rash presentation with associated symptoms, as well as the progression of symptoms in succession to the completion of azithromycin and doxycycline.

## Discussion

SJS is a rare, acute, and potentially fatal severe skin reaction where medications are the causative trigger in 80% of cases. If greater than 30% of the skin is affected, it is classified as TEN. SJS/TEN may present with general malaise and an upper respiratory tract infection, along with a rash that is erythematous and targetoid in appearance. Patients will additionally show purpuric macules and flaccid bullae and have a positive Nikolsky sign in most but not all cases. The exact etiology of SJS/TEN is unknown. However, it is theorized that it is due to a T-cell-mediated pathway where CD8+ T cells induce keratinocyte apoptosis. A punch biopsy will reveal “necrosis of keratinocytes, epidermal (or epithelial) necrosis, and mild lymphocytic dermal infiltration. Direct immune fluorescence is negative” [3]. 

Azithromycin, along with erythromycin and clarithromycin, is a macrolide and is overall considered safe due to its few adverse reactions. There have been some reported cases of QTc prolongation, but azithromycin remains relatively less cardiotoxic when compared to the other macrolides. It is much more commonly associated with gastrointestinal adverse reactions such as nausea, vomiting, diarrhea, and abdominal pain/discomfort [4]. As mentioned before, FDA labeling indicates azithromycin as a cause of SJS/TEN, stating that "serious allergic reactions, including angioedema, anaphylaxis, and dermatologic reactions including Stevens-Johnson syndrome and TEN have been reported in patients on azithromycin therapy" [5]. Of all of the macrolide antibiotics, azithromycin accounts for the majority of reported SJS/TEN cases (11 of 27 cases in one study), with time to onset ranging from one to 14 days with a median of three days [6]. However, macrolides overall represent only 2% of antibiotic-associated SJS/TEN cases in meta-analysis [1].

Doxycycline is a tetracycline that comes with common adverse reactions such as photosensitivity, skin rash, urticaria, and fixed drug eruptions [5]. Some severe cases of adverse reactions due to the drug include exacerbation of systemic lupus erythematosus and, in rare cases, SJS. Doxycycline has also been identified as a significant risk factor for SJS/TEN. A recent study analyzing the FDA Adverse Event Reporting System found that doxycycline exhibited a positive risk signal for SJS/TEN through disproportionality analysis [7]. Overall, it is difficult to conclude which antibiotic caused the skin reaction due to the quick succession of administration of both medications.

Mortality rates range from 1% to 5% for SJS to 25% to 35% for TEN, further indicating that prompt recognition and withdrawal of the triggering medication can be critical for patient survival [8]. Treatment involves a multidisciplinary approach with supportive care in specialized units, immediate discontinuation of suspected medications, and consideration of immunomodulatory therapies [9].

The patient's advanced age (76 years) represents an additional risk factor. Older adults have increased susceptibility to serious cutaneous adverse drug reactions due to polypharmacy, comorbidities, and age-related physiologic changes [3]. The median age for SJS is 63 years and for TEN is 60 years, suggesting increased incidence with advancing age, creating a risk factor for the patient in this case [7]. Sequential exposure to two potential culprit antibiotics complicates attribution in this case. The development of the rash occurring shortly after completing both antibiotics makes it difficult to definitively identify which of the two drugs triggered the reaction [3,10].

This case illustrates the diagnostic challenges associated with cutaneous adverse drug reactions, particularly when histopathologic findings are nonspecific. This patient was known to have completed courses of azithromycin and doxycycline [11]. In this patient, preliminary skin biopsy results demonstrated benign skin with mild perivascular chronic inflammation, a finding that lacks specificity and is thus nonconclusive. However, final pathology results later characterized the specimen as a dermal hypersensitivity reaction, supporting a drug-induced etiology [12]. These biopsy findings, when interpreted in conjunction with the patient’s clinical presentation, timeline of antibiotic exposure to symptom presentation, systemic inflammation, and subsequent improvement following discontinuation of the suspected agents and initiation of systemic corticosteroids, strongly suggest a severe medication-related hypersensitivity reaction. Although the biopsy findings were nonspecific, this case may represent a severe drug-induced hypersensitivity reaction rather than confirmed Stevens-Johnson syndrome. This case highlights the importance of correlating histopathologic results with clinical context, as early biopsy specimens in severe drug eruptions may be subtle or nondiagnostic. Prompt recognition, withdrawal of antibiotics, and initiation of supportive and anti-inflammatory therapy remain the cornerstone of management and may prevent progression to more severe cutaneous syndromes such as SJS or TEN.

## Conclusions

In most cases, SJS/TEN patients should be admitted for close supervision and management due to the greater risk for complications of the reaction. Assessment through interprofessional consultations, such as dermatology, an intensivist, a respiratory physician, and a burn specialist, is highly recommended. The exact treatment varies depending on the severity of SJS/TEN. A mild eruption will typically take approximately 12-16 weeks to completely heal. 

In this case, definitive histological findings were still pending at the time of the patient’s symptom resolution and discharge. This imposed a challenge on the approach to care, as a clinical rather than pathological diagnosis had to be made. As there is a greater amount of literature on medication-induced dermal reactions leading to SJS, management was approached carefully through the interplay of multiple specialties to prevent any complications or worsening of symptoms. In addition, there is a greater risk of doxycycline causing rashes as an adverse reaction compared to azithromycin. However, moving forward, the patient has been advised to avoid both azithromycin and doxycycline for antibiotic use. Due to the consecutive use of antibiotics just prior to the eruption of the rash, it is difficult to identify the exact root cause of the reaction. Although the biopsy findings were nonspecific, this case may represent a severe drug-induced hypersensitivity reaction rather than confirmed SJS. However, due to the risk of a more severe reaction occurring with a second exposure, using an alternative antibiotic with caution was advised. 
